# Case report of schizophrenia in a patient with de novo mutation in the SLC6A1 gene

**DOI:** 10.1192/j.eurpsy.2023.1886

**Published:** 2023-07-19

**Authors:** V. Mikhailova, M. Alfimova, T. Lezheiko, N. Kondratyev, V. Kaleda, A. Galiakberova, E. Dashinimaev, V. Golimbet

**Affiliations:** 1 Clinical Genetics Laboratory, Mental Health Research Center; 2 Center for Precision Genome Editing and Genetic Technologies for Biomedicine, Pirogov Russian National Research Medical University, Moscow, Russian Federation

## Abstract

**Introduction:**

Schizophrenia is a severe mental disorder mainly caused by genetic risk factors. Many studies have demonstrated that both multiple genetic variants and rare mutations are associated with schizophrenia risk. The next step is to study the causal effect of the gene on the phenotype. Recently, a large family-based study identified de novo mutations, which may increase liability to schizophrenia (Rees et al 2020). In particular, a mutation in the GABA transporter (SLC6A1) gene (rs756927822 C/T) was identified in one patient from our subsample.

**Objectives:**

Here, we present a case report of this patient and describe the procedure of derivation of induced pluripotent stem cells (iPSCs) from fibroblast cultures.

**Methods:**

Clinical, psychometric and neuropsychological methods were used. iPSCs were derived from patients’ and both unaffected parents’ fibroblasts. Human fibroblasts, cultured in fibroblast medium, are infected with lentivirus vectors expressing the transcription factors Oct4, Sox2, c-Myc, and KLF4. All iPSCs were immunocytochemical stained for intracellular (Oct4, Sox2) and extracellular (SSEA4, Tra-1-81, Tra-1-60) pluripotency markers. An qPCR analysis for pluripotency markers (TDGF1, Sox2, Oct4, REX1, LIN28, NANOG, KLF4, GDF3, DPPA4, DNMT3) was performed. All four iPSC lines formed embryoid bodies before the differentiating into three germ layers. Differentiation was confirmed by immunostaining for mesoderm (aSMA), ectoderm (Nestin, Desmin) andendoderm (FoxA2, Pax6) markers.

**Results:**

A 47-year-old male patient was presented to psychiatry at the age of 16. There was no personal or family history of psychiatric disorder, the premorbid functioning was normal, the patient had no somatic diseases, showed high performance in sport (mountain skiing). On his first admission, he was diagnosed with schizoaffective psychosis. The patient showed signs of mania and catatonia. Neuropsychological testing revealed a decrease of cognitive functioning (short-term and associative memory). The patient was followed up for more than 20 years. The diagnosis was changed for schizophrenia at the age of 43 years. There was a deterioration in cognitive function (the apparent decrease in performance on neurocognitive tests (attention, memory, executive functions) from the first examination (1997) till last one (2019). The patient refused or was not able to perform most of the tasks. During follow-up, the patient shows good adherence to treatment.

**Conclusions:**

For this patient, obtained lines might be valuable for investigating the disease mechanisms and screening candidate drugs.

**Disclosure of Interest:**

None Declared

